# Late dumping syndrome preceded by Coxsackievirus B4 infection and cholecystectomy

**DOI:** 10.1093/jscr/rjad205

**Published:** 2023-04-25

**Authors:** Joshua Weiss, Connor Fewel, Oyediran Akinrinade, Jane Harrington

**Affiliations:** Department of Microbiology, St. George’s University, School of Medicine, True Blue Campus, St. George, Grenada, West Indies; Department of Microbiology, St. George’s University, School of Medicine, True Blue Campus, St. George, Grenada, West Indies; Department of Microbiology, St. George’s University, School of Medicine, True Blue Campus, St. George, Grenada, West Indies; Department of Microbiology, St. George’s University, School of Medicine, True Blue Campus, St. George, Grenada, West Indies

## Abstract

A 44-year-old female patient presented with weight loss, diarrhea and intermittent episodes of left upper quadrant (LUQ) pain lasting for 3 years, accompanied by acute episodes of focal LUQ pain, dizziness, tachycardia, borborygmi and bloating, occurring approximately 60 min after meals. The patient developed chronic acalculous cholecystitis and transient exocrine pancreatic insufficiency after infection with Coxsackievirus B4 (CVB4), which resolved following laparoscopic cholecystectomy 2 years before the current presentation. Although imaging and functional investigation studies were unremarkable, a gastric transit study revealed rapid clearance of radiolabeled food, and the patient’s symptomatology and gastrointestinal studies supported the diagnosis of late dumping syndrome. The patient’s symptoms significantly improved with adherence to recommended dietary changes, including an increase in protein intake, abstinence from simple carbohydrates and avoidance of simultaneous consumption of beverages with food, following consultation with a dietitian.

## INTRODUCTION

Dumping syndrome (DS) is a condition characterized by the accelerated passage of hyperosmolar contents from the stomach to the small bowel [1, 2]. It presents as early DS with symptoms occurring within 30 min of a meal or late DS with onset occurring 1–3 h after consuming a meal, triggering postprandial hyper-insulinemic hypoglycemia. While DS is frequently associated with bariatric surgery, approximately 30% of patients have no identifiable trigger [2, 3]. Patients who undergo subsequent cholecystectomy after bariatric surgery have a higher risk of developing DS [4]. Cholecystectomy may contribute to the risk of developing DS [5], particularly in combination with other factors that alter gastrointestinal function.

DS severity was once thought to be linked to the extent of gastric surgery. However, nonsurgical risk factors for DS have been identified, such as vagal nerve damage, pancreatic dysfunction and complications of diabetes, as well as viral infections including SARS-CoV2 and Enteroviruses. These infections have been implicated as triggers for pancreatitis and gastrointestinal motility disorders [2, 6, 7]. Coxsackievirus serotype B4 (CVB4), which binds to host coxsackievirus and adenovirus receptors (CARs) and decay-accelerating factor (DAF) receptors, has been shown to cause localized damage to various cells including intestinal epithelial, β-islet, pleural muscular and neuronal cells, leading to diverse clinical manifestations. In a previous report, we linked CVB4 infection to chronic acalculous cholecystitis [8]. This case study presents a follow-up complication of late DS following CVB4 infection and cholecystectomy.

## CASE REPORT

**Figure 1:**
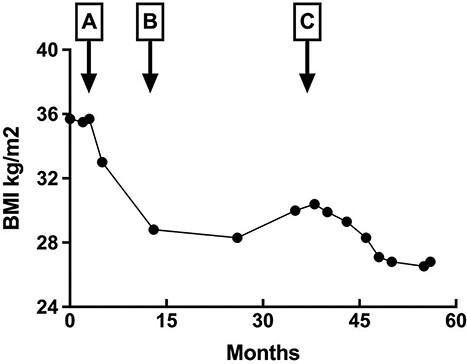
Change in body mass index (BMI). Change in patient body mass index BMI (height 152.4 cm) corresponding to inflammatory insults indicated with arrows: (**A**) Coxsackievirus B4 infection; (**B**) cholecystectomy and (**C**) pyelonephritis and bronchopneumonia following 5-month course of immunosuppressants. Patient maintained a predominantly plant-based diet without intentional restriction. Periods of rapid weight loss corresponded with the duration of persistent abdominal pain and diarrhea.

A 44-year-old Caucasian female, G1P1, presented to a gastroenterology clinic with a 6-month history of diarrhea and unintentional weight loss. She had sporadic episodes of acute LUQ pain, accompanied by dizziness, flushing, tachycardia and exhaustion that occurred 45–90 min after ingesting food and beverages over a three-year period. The patient had a history of chronic acalculous cholecystitis and transient pancreatic insufficiency due to infection with CVB4, which led to cholecystectomy at age 41 (Fig. 1A and B). Additionally, the patient had a diagnosis of post-viral seronegative inflammatory arthritis and fibromyalgia, following an infection with Chikungunya virus at age 36, associated with spinal pain which lasted for 10 months. The patient was previously on methotrexate and sulfasalazine but discontinued the medications after developing bronchopneumonia and pyelonephritis 4 months prior to the current presentation, coinciding with the onset of weight loss, abdominal pain, diarrhea and increased frequency of hypoglycemic episodes (Fig. 1C).

During the patient’s visit to the gastroenterology clinic, vital signs were normal, and the patient had a BMI of 29.3 kg/m^2^. Hematology and metabolic studies were normal, except for a mild elevation in absolute eosinophil counts (0.6 × 10^9^ cells/L). The patient’s fasting glucose level was 94 mg/dL, and HbA1c was 4.3%. The patient displayed a Type-I hypersensitivity reaction to 80% of the environmental allergens tested. Various gastrointestinal studies, including enteroscopy, MRI, esophagogastroduodenoscopy (EGD), colonoscopy and anorectal manometry, revealed no structural or functional abnormalities. Pathology studies of EGD samples of the antral and fundic mucosa indicated reactive gastropathy. The gastric emptying scintigraphy transit study revealed rapid emptying at 2 h (Table 1), leading to the diagnosis of late DS based on the patient’s clinical history, symptomatology and GI transit study results. The patient was subsequently referred to a nutritionist for dietary and behavioral modifications, resulting in weight stabilization and resolution of gastrointestinal symptoms.

**Table 1:** Gastric emptying scintigraphy transit study patient consumed toast, egg omelet and 250 mL milk radiolabeled with ^99m^Tc-SC and gastric emptying was monitored with an image taken on a scintillation camera at indicated time points. Gastric emptying was high normal at 1 h, rapid at 2 h, indicated with ^*^, normal at time points 0 and 4 h

	Patient	Normal range
Emptying at 0 h	0	0
Emptying at 1 h	20.1	7–27%
Emptying at 2 h	88^*^	31–67%
Emptying at 4 h	99.7	81–100%

## DISCUSSION

DS is a multifactorial condition with a unique pathophysiology for each patient. Patients with multiple aggravating factors causing inflammation of the gastrointestinal tract (GIT) present with DS at an increased rate [4]. In this case study, the patient experienced abdominal pain and rapid weight loss associated with CVB4 infection, leading to pleurodynia and acalculous cholecystitis (Fig. 1A and B). Symptoms resolved post-cholecystectomy, and the patient maintained a stable BMI with adherence to a healthy lifestyle. The patient experienced increased frequency of dumping episodes and unintentional weight loss after concurrent infections while on immunosuppressants (Fig. 1C), and the gastric transit study was grossly abnormal at 120 min (Table 1), which yielded the diagnosis of dumping syndrome.

CVB4 is known to establish a carrier state in which it continues to cause variable systemic insults. The timeline of abdominal pain and weight loss implicates chronic inflammation triggered by infection, which is further supported by the development of pleurodynia, pancreatic insufficiency, acalculous cholecystitis [8] and progressive DS. The established retrograde nerve travel of Enteroviruses [9] and the myenteric plexus connecting affected organs lead to a plausible mechanism for the onset of DS. Inflammation caused by cholecystectomy prior to the onset of DS is consistent with findings of approximately 56% of patients developing DS after cholecystectomy with a history of prior GIT insults [10].

Hyperglycemia, a known factor associated with gastroparesis [2], can lead to DS by activating mitogen-activated protein kinases (MAPKs) and subsequently receptor tyrosine kinase (KIT) in the interstitial cells of Cajal (ICCs), resulting in hypertrophy and proliferation [11] Activated p38 MAPKs play an integral role in cell survival, proliferation, inflammation and stress responses [12]. The ICC is the pacemaker cell of the GIT, thus chronic activation can result in dumping syndrome. In this case report, the initial CVB4 infection likely activated similar physiological pathways as hyperglycemia, resulting in hypertrophy of the ICC. Reactivation of latent CVB4 infection and inflammation could have led to the proliferation of the ICC. Cholecystectomy in the patient also contributed to the activation of MAPKs by causing local inflammation.

The course of methotrexate and sulfasalazine resulted in immunosuppression, likely exacerbating the damaging effects of the CVB4 virus. The patient experienced precipitous weight loss concurrent with an increased frequency of dumping episodes (Fig. 1, arrow C). The manifestation of multisystem inflammation indicates worsening disease severity due to the immunocompromised state secondary to the effects of the pharmaceutical interventions.

This case report shows that the risk of DS increases as the number of GI insults accumulates, including gastrointestinal surgery following CVB4 infection, a novel contributing risk factor for DS. As medical professionals gain knowledge of the risk factors involved in DS pathophysiology, they can better identify, diagnose and intervene early in this condition.

## Data Availability

Data is available for review upon request.
